# Lanthanide-based metal–organic frameworks solidified by gelatin-methacryloyl hydrogels for improving the accuracy of localization and excision of small pulmonary nodules

**DOI:** 10.1186/s12951-022-01263-6

**Published:** 2022-02-02

**Authors:** Haoran Ji, Xiaofeng Wang, Pei Wang, Yan Gong, Yun Wang, Chang Liu, Guangyu Ji, Xiansong Wang, Mingsong Wang

**Affiliations:** 1 Department of Thoracic Surgery, Shanghai Key Laboratory of Tissue Engineering, Shanghai Ninth People’s Hospital, Shanghai Jiao Tong University School of Medicine, Shanghai, 200011 China; 2 Excellent Science and Technology Innovation Group of Jiangsu Province, College of Environmental Science, Nanjing Xiaozhuang University, Nanjing, 211171 China

**Keywords:** Lanthanide-based metal–organic framework, Gelatin-methacryloyl, Localization of small pulmonary nodules, Dual-modal imaging

## Abstract

The localization of invisible and impalpable small pulmonary nodules has become an important concern during surgery, since current widely used techniques for localization have a number of limitations, such as invasive features of hookwires and microcoils, and rapid diffusion after injection of indocyanine green (ICG). Lanthanide-based metal–organic frameworks (MOFs) have been proven as potential fluorescent agents because of their prominent luminescent characteristics, including large Stokes shifts, high quantum yields, long decay lifetimes, and undisturbed emissive energies. In addition, lanthanides, such as Eu, can efficiently absorb X-rays for CT imaging. In this study, we synthesized Eu-UiO-67-bpy (UiO = University of Oslo, bpy = 2,2'-bipyridyl) as a fluorescent dye with a gelatin-methacryloyl (GelMA) hydrogel as a liquid carrier. The prepared complex exhibits constant fluorescence emission owing to the luminescent characteristics of Eu and the stable structure of UiO-67-bpy with restricted fluorescence diffusion attributed to the photocured GelMA. Furthermore, the hydrogel provides stiffness to make the injection site tactile and improve the accuracy of localization and excision. Finally, our complex enables fluorescence-CT dual-modal imaging of the localization site.

**Supplementary Information:**

The online version contains supplementary material available at 10.1186/s12951-022-01263-6.

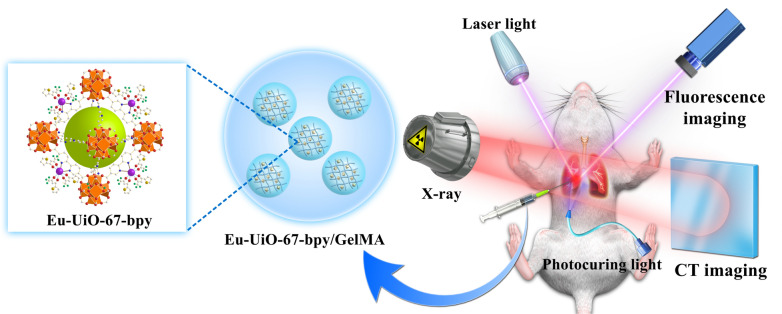

## Introduction

The early diagnosis of lung cancer has been effectively promoted in the past few years due to the widespread use of low-dose computed tomography (CT) [1–3]. Using CT, the diagnosis and treatment of small pulmonary nodules (i.e., lesions with diameters < 1 cm, which are related to benign disease or early-stage lung cancer) have received growing attention from medical experts [4, 5]. However, the localization of small pulmonary nodules can be challenging because the lesions are often invisible and impalpable during surgery, and collapse of the lung can also make localization more difficult [6].

Thus, a number of preoperative tumor marking techniques have been developed to facilitate the localization of small pulmonary nodules, including the use of hookwires, microcoils, and dyes. Hookwires and microcoils are widely used as metal tags for localization. However, their invasion features can cause complications, including patient discomfort, pneumothorax, and hemothorax. Dislodgement of the tags may also occur due to the movement associated with respiration [7]. In contrast, indocyanine green (ICG), a dye that can be visualized by near-infrared (NIR) fluorescence imaging, is also commonly employed in the localization of pulmonary nodules because of its safety and NIR emission spectrum, which provides a fine penetrability and avoids the autofluorescence of any undyed tissue [8, 9]. Nevertheless, as a small-molecule fluorescent dye, ICG is unstable, and is easily quenched under protracted laser excitation [10]. Furthermore, rapid diffusion of the ICG solution into the surrounding pulmonary parenchyma and visceral pleura may decrease the accuracy of localization [11]. Moreover, ICG is difficult to observe in CT images prior to surgery when it is necessary to confirm the localization site, although it can be observed immediately after injection. As a result, surgery must be performed soon after the injection of ICG, which can complicate arrangement of the preoperative localization process, in addition to the surgery itself. ICG is also not suitable for deep localization because of the limited penetration depth of NIR irradiation beyond 10 mm, despite the fact that the penetrability of NIR is considered relatively strong compared to other types of irradiation [12].

Accordingly, despite the numerous merits and wide use of ICG, there remains a necessity to develop new fluorescent species or their combinations to improve preoperative localization.

In this context, owing to its large Stokes shift, narrow emission wavelength, and long luminescence lifetime, europium (Eu^3+^) has attracted particular interest in luminescence applications [13–15]. Eu^3+^ presents high K-edge values and X-ray coefficients because Eu has a high atomic number, and so Eu^3+^ efficiently absorbs X-rays for CT imaging [16]. Therefore, these physical characteristics render it possible for materials containing Eu^3+^ to achieve dual-modal imaging. However, the practical applications of Eu complexes have been limited by a number of inherent drawbacks, such as their poor mechanical and unrecoverable properties, as well as quenching due to aggregation [17]. To overcome these limitations, doping Eu^3+^ into host materials to produce hybrid complexes has been found to be an effective strategy [18]. Fortunately, with the development of nanotechnology and materials science, several designs have been proposed, including the utilization of metal–organic frameworks (MOFs). MOFs are organic–inorganic hybrid materials constructed from metal ions or metal ion clusters and bridging organic linkers, which have drawn increased attention during the past few decades [19]. Owing to their tailorable porous channels, stable frameworks, and rich active sites, MOFs can accommodate and modify guest molecules to achieve various functions [20–23]. In addition, due to their outstanding biosecurity ensured by a good dispersibility and biocompatibility, MOFs have been employed in biomedical applications, including fluorescent probes and optical imaging [24]. Lanthanide MOFs, especially Eu(III)-MOFs, which exhibit unique luminescent properties, including large Stokes shifts, high quantum yields, long decay lifetimes, and undisturbed emissive energies, are considered to be promising fluorescent materials for use in various fields [25–27]. In our previous work, we found that Mg-MOFs possess excellent biological functions that could be promising for their application in drug delivery [28]. However, the Mg-MOFs were easily degraded, and the fluorescence was not stable upon the incorporation of Eu^3+^ in our previous attempts. Thus, we adjusted our scheme and designed a fluorescent dye by loading lanthanide Eu^3+^ cations onto frameworks of UiO-67-bpy (UiO = University of Oslo, bpy = 2,2'-bipyridyl), which are a type of zirconium-based MOF, and as a result, we obtained a satisfactory fluorescence. Zr-MOFs have recently received increasing attention, as they have been proven to be thermally, mechanically, and chemically stable [29, 30], in addition to possessing a good biocompatibility for biomedical applications [31]. Besides, Zr-MOFs are excellent platforms for postsynthetic modification, which can allow various functions to be achieved [32]. For example, Eu post-functionalized Zr-MOFs have been proved to be effective sensitive fluorescent probe for bilirubin [33]. Among the various types of Zr-MOFs reported to date, UiO-67-bpy has been widely investigated owing to the rich coordination chemistry of its 2,2'-bipyridine (BPY) moiety, which can facilitate the combination of various ligand types, including metal complexes [34]. Furthermore, 2,2'-bipyridine is one of the most widely used chelating ligands for developing metal complexes [35]. When employed in lanthanide complexes, such as those of Eu, 2,2'-bipyridine is able to photosensitize the lanthanide ion through intramolecular energy transfer to overcome the weak absorption of lanthanides resulting from the forbidden character of the 4f–4f transitions [17].

Although this Eu-MOF (Eu-UiO-67-bpy) could provide a solution for the bleaching of fluorescence, such as that occurring in the case of ICG, the issue related to the rapid diffusion of the fluorescence dye within the tissue must be addressed for clinical applications. We therefore considered the adoption of a gelatin-methacryloyl (GelMA) hydrogel as a liquid carrier. GelMA hydrogels, which are biocompatible, biodegradable, non-cytotoxic, and non-immunogenic, have been widely used for many applications ranging from tissue engineering to drug and gene delivery [36–38]. These hydrogels undergo photoinitiated radical polymerization and become covalently crosslinked under visible light or ultraviolet (UV) light exposure in the presence of a photoinitiator [39]. When carrying fluorescent nanoparticles, crosslinked hydrogels can achieve controlled material release to maintain the fluorescence for a longer period of time, which could also reduce the potential cytotoxicity of materials to normal parenchyma. Furthermore, hydrogels can provide additional stiffness to render the complex more tactile, which is of significance in the localization of unpalpable pulmonary nodules.

**Scheme 1:**
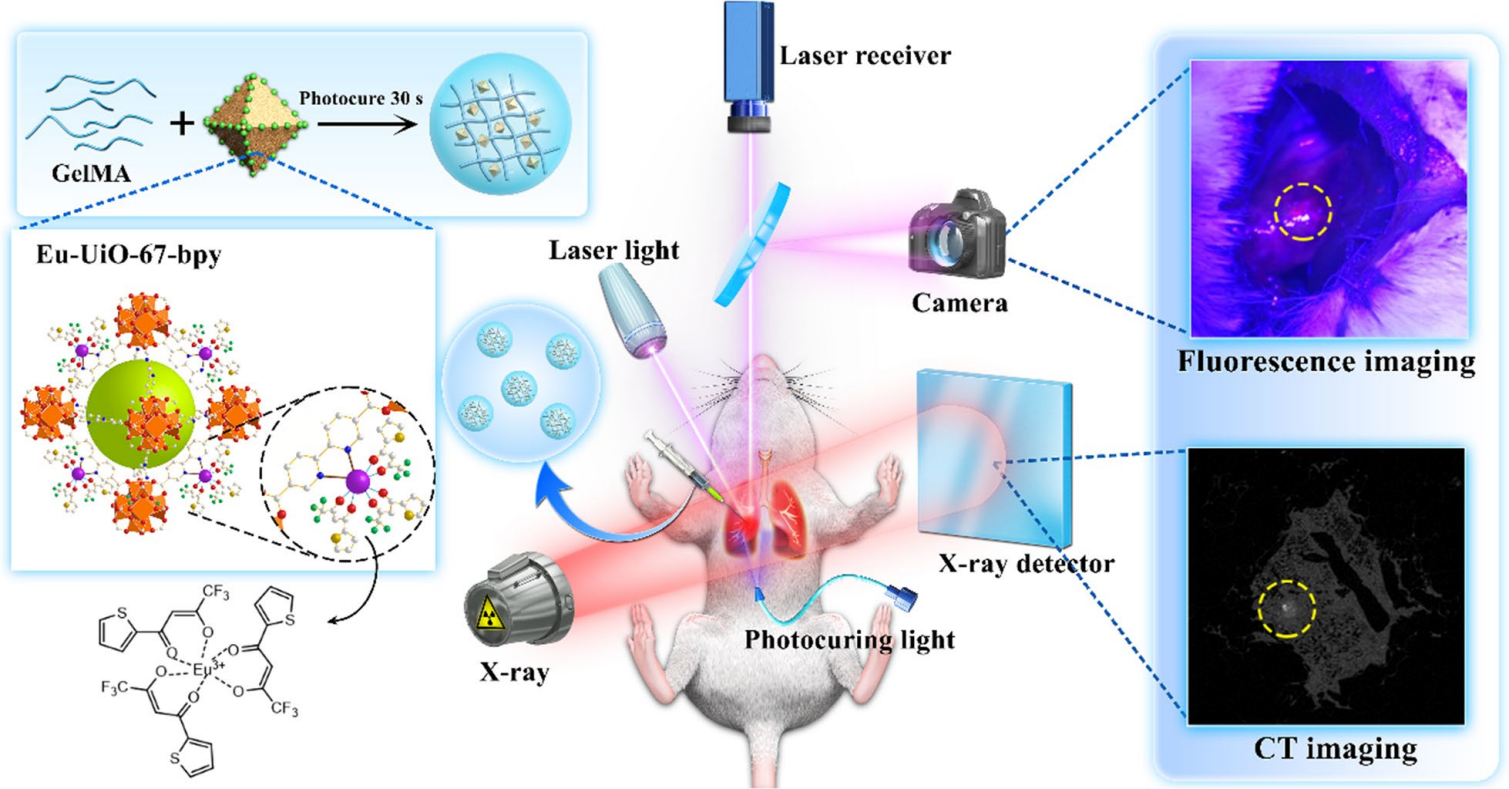
Schematic illustration of the components of the Eu-MOF/GelMA composite hydrogel and its application in fluorescence/CT dual-modal imaging

Based on the reported characteristics of the material components described above, we report the preparation of a Eu-MOF/GelMA complex with the aim of refining the localization of small pulmonary nodules. A range of in vitro and in vivo experiments are also designed and carried out to verify the applicability of our system in this context (Scheme 1). We expect that the fluorescence emission could be more constant owing to the luminescent characteristics of Eu and the stable structure of UiO-67-bpy. In addition, the GelMA component has the potential to inhibit the diffusion of fluorescent dyes and to render the injection site more tactile to ultimately improve the accuracy of localization and excision. Moreover, our complex could enable the dual-modal imaging of the surgical target, including both CT and fluorescence imaging.

## Results

### Fabrication and characterization of the Eu-MOF and the Eu-MOF/GelMA composite hydrogel

**Fig. 1:**
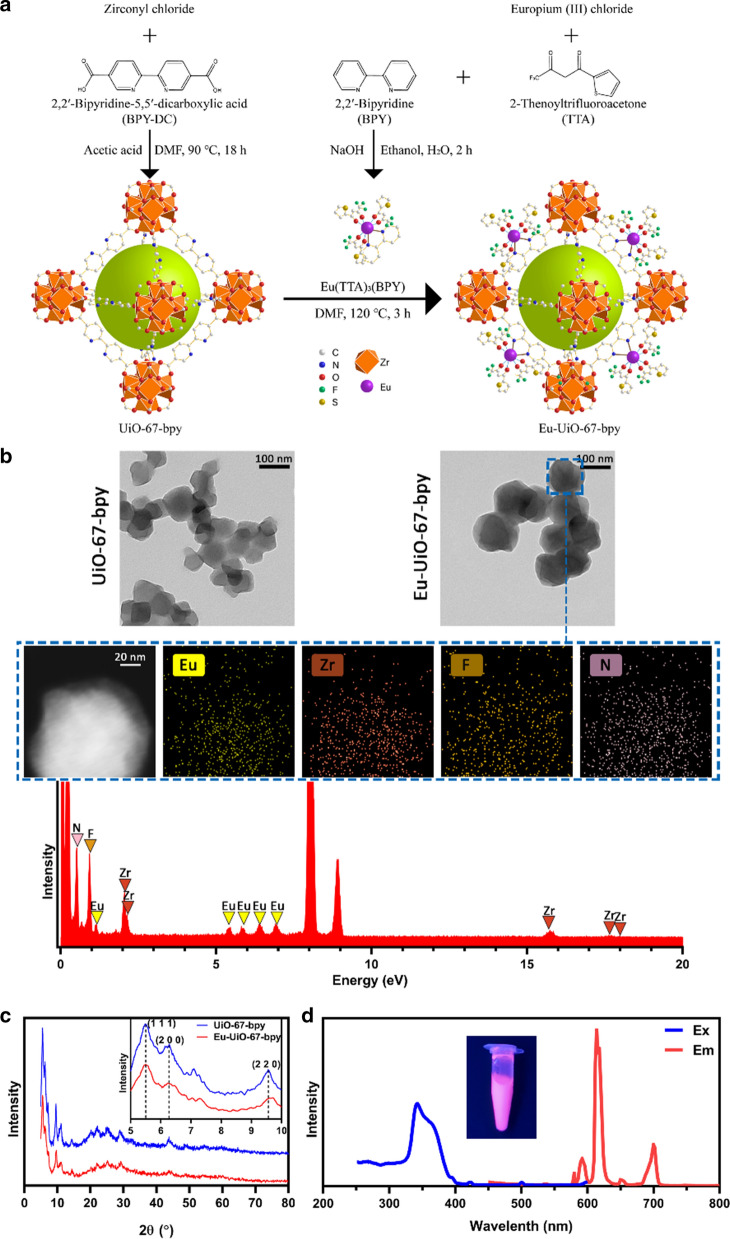
Fabrication and characterization of the Eu-MOF. **A** Schematic diagram outlining the synthesis of the Eu-MOF. **B** TEM and mapping images of the Eu-MOF. **C** XRD pattern of the Eu-MOF. **D** Excitation (Ex, blue line) and emission (Em, red line) spectra of the Eu-MOF. The inset shows a suspension of the fluorescent Eu-MOF under 365 nm UV light excitation

As shown in the synthetic route outlined in Fig. 1A, the UiO-67-bpy MOF nanoparticles and the Eu(TTA)_3_(BPY) fluorescence imaging agent (TTA, 2-thenoyltrifluoroacetone, BPY, 2,2'-bipyridine) were synthesized and then mixed together at 120 °C to fix the fluorescence agent onto the MOF structure via a ligand-exchange reaction. Figure 1B shows the transmission electron microscopy (TEM) images of the UiO-67-bpy and Eu-UiO-67-bpy species, wherein the diameter of Eu-UiO-67-bpy was determined to be 100–120 nm. The element mapping images indicate that Eu, Zr, F, and N were uniformly distributed within the framework of the MOF. X-ray diffraction (XRD) experiments also confirmed the successful formation of the MOF structure of Eu-UiO-67-bpy (Fig. 1C). As shown in the photoluminescence excitation and emission spectra (Fig. 1D), the excitation and emission peaks were observed at 342 and 614 nm, respectively, thereby indicating that the suspension of Eu-UiO-67-bpy can emit strong fluorescence upon irradiation with UV light. Moreover, the suspensions of GelMA, Eu-MOF/H_2_O, and Eu-MOF/GelMA were formed as fine dispersions, as verified by the Tyndall effect (Additional file 1: Fig. S1).

**Fig. 2:**
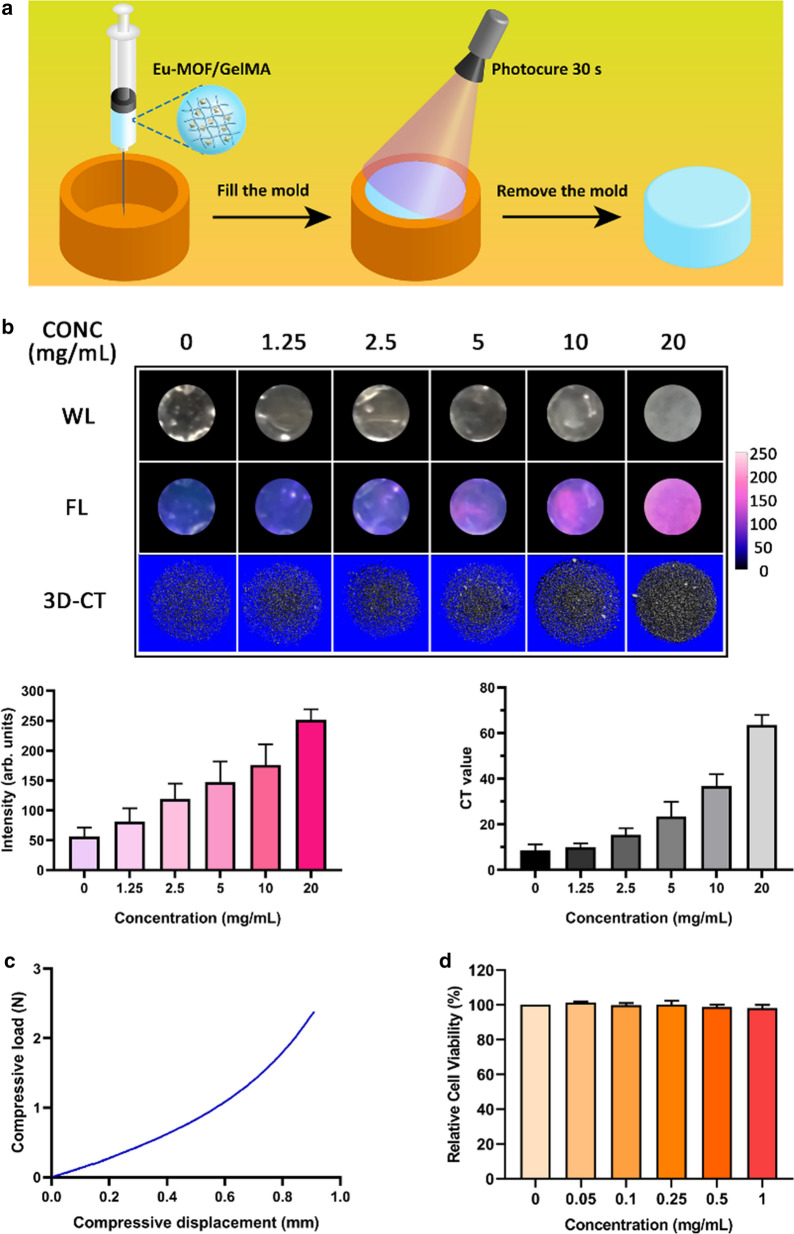
Characterization of the Eu-MOF/GelMA composite hydrogel. **A** Preparation of the Eu-MOF/GelMA composite hydrogel block. **B** Fluorescence images and 3D reconstructions of the CT images of the Eu-MOF/GelMA composite hydrogel with Eu-MOF concentrations ranging from 0 to 20 mg/mL. The corresponding fluorescence intensities and CT values are given in the plots below the images. **C** Compressive load-compressive displacement curve of the Eu-MOF/GelMA composite hydrogel with an Eu-MOF concentration of 20 mg/mL. **D** Relative cell viability determined by a CCK-8 assay of HFL1 cells incubated with different concentrations of the Eu-MOF for 24 h

Figure 2A shows the preparation process employed to obtain the Eu-MOF/GelMA composite hydrogel blocks. Fluorescence images and a three-dimensional (3D) reconstruction of the CT images of the Eu-MOF/GelMA composite hydrogel blocks are shown in Fig. 2B, wherein the relative fluorescence intensities (arb. units) of different hydrogel concentrations (i.e., 0, 1.25, 2.5, 5, 10, and 20 mg/mL) were 56.152 ± 14.933, 81.303 ± 22.294, 119.060 ± 25.916, 147.305 ± 34.401, 176.172 ± 34.430, and 251.489 ± 17.709, respectively. These results indicate that a higher Eu-MOF concentration led to an increased fluorescence intensity. In addition, the corresponding CT values (HU) were 8.550 ± 2.584, 10.039 ± 1.572, 15.338 ± 3.018, 23.521 ± 6.399, 36.805 ± 5.136, and 63.547 ± 4.418, respectively. It was also observed that the hydrogel with the highest concentration showed a significantly higher density in the 3D reconstruction images compared to those obtained in the absence of the Eu-MOF. Furthermore, the stiffness of the Eu-MOF/GelMA composite hydrogel was measured using the compressive modulus. According to the compressive load–displacement curve (Fig. 2C), the compressive modulus of the complex was 0.13725 ± 0.01120 MPa, which was sufficiently high to be distinguished from soft tissues (e.g., pulmonary parenchyma) by finger touch alone (see Additional file 3: Video S1). The biocompatibility of the Eu-MOF was then evaluated using the Cell Counting Kit-8 (CCK-8) assay, as shown in Fig. 2D. More specifically, HFL1 cells were incubated with the Eu-MOF at concentrations ranging from 0 to 1 mg/mL for 24 h. The CCK-8 assay revealed a relative cell viability of > 98% for various Eu-MOF concentrations [i.e., (101.32 ± 0.65)% for 0.05 mg/mL, (99.83 ± 1.19)% for 0.1 mg/mL, (100.24 ± 2.12)% for 0.25 mg/mL, (98.78 ± 1.35)% for 0.5 mg/mL, and (98.18 ± 1.82)% for 1 mg/mL].

### In vitro study

**Fig. 3:**
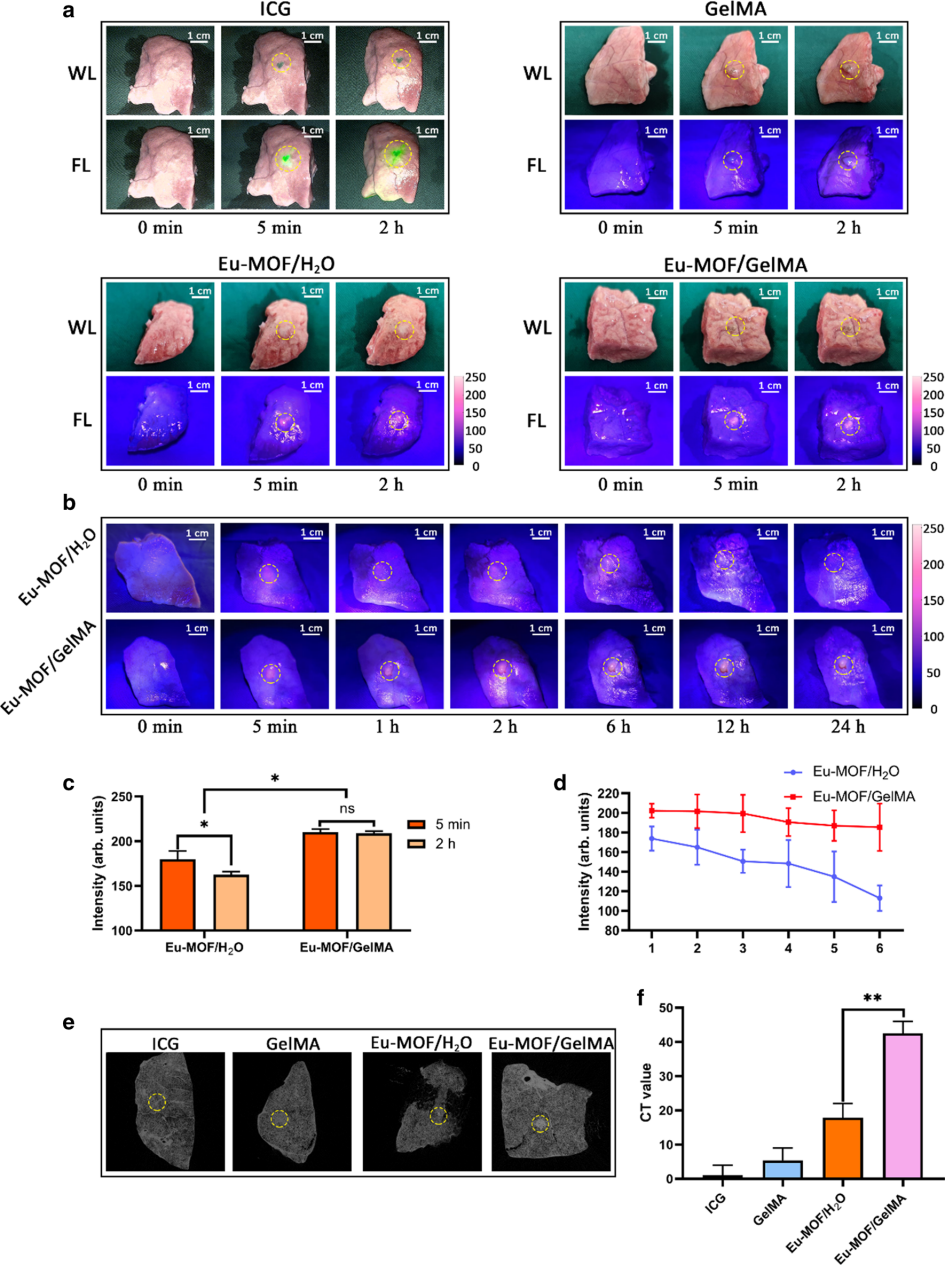
In vitro study of dual-modal imaging. **A** Fluorescence images of a 2-h observation of porcine lung segments injected with an ICG aqueous solution, a GelMA solution, a Eu-MOF/H_2_O suspension, and a Eu-MOF/GelMA suspension. **B** Fluorescence images of a 24-h observation of porcine lung segments injected with Eu-MOF/H_2_O and Eu-MOF/GelMA suspensions. **C** Fluorescence intensities of the injection sites of the porcine lung segments injected with Eu-MOF/H_2_O and Eu-MOF/GelMA suspensions (5 min and 2 h after injection). **D** Fluorescence intensity variations of a 24-h observation of porcine lung segments injected with Eu-MOF/H_2_O and Eu-MOF/GelMA suspensions. **E** CT images of porcine lung segments taken 2 h after injection with an ICG aqueous solution, a GelMA solution, a Eu-MOF/H_2_O suspension, and a Eu-MOF/GelMA suspension. **F** CT values of the injection sites of porcine lung segments. *P < 0.05, **P < 0.01

As shown in Fig. 3A, the ICG diffused rapidly into the surrounding pulmonary parenchyma of the porcine lung segments during the 2 h immediately after injection. In the case of the Eu-MOF/H_2_O combination, the luminance of the fluorescent area reduced significantly after this time, and the border between the fluorescent area and the normal lung tissue became more obscure (Fig. 3A, C). However, the Eu-MOF/GelMA composite hydrogel maintained both a restrictive fluorescent area and a high fluorescence intensity after 2 h.

In addition, as shown in Fig. 3B and D, we observed variations in the fluorescent areas of the Eu-MOF/H_2_O and Eu-MOF/GelMA samples over 24 h. More specifically, after injection, the Eu-MOF/H_2_O suspension diffused immediately along the lung surface, and as time progressed, the fluorescence intensity decreased. After 6 h, only a dim fluorescent dot was observed at the injection point, and after 24 h, the fluorescence became almost invisible. In contrast, the fluorescence of the Eu-MOF/GelMA system was strong and stable, with little change taking place during the 24-h period. Indeed, the fluorescence was maintained even after 48 and 72 h (Additional file 2: Fig. S2). CT images also showed obvious differences among the ICG, GelMA, Eu-MOF/H_2_O, and Eu-MOF/GelMA samples (Fig. 3E), with CT values (HU) of 1.046 ± 2.954, 5.456 ± 3.544, 17.913 ± 4.157, and 42.601 ± 3.432 being determined, respectively (Fig. 3F). Moreover, the injection site of the Eu-MOF/GelMA hydrogel showed an apparent increase in its CT value, which could be easily observed by CT imaging.

### In vivo study

**Fig. 4:**
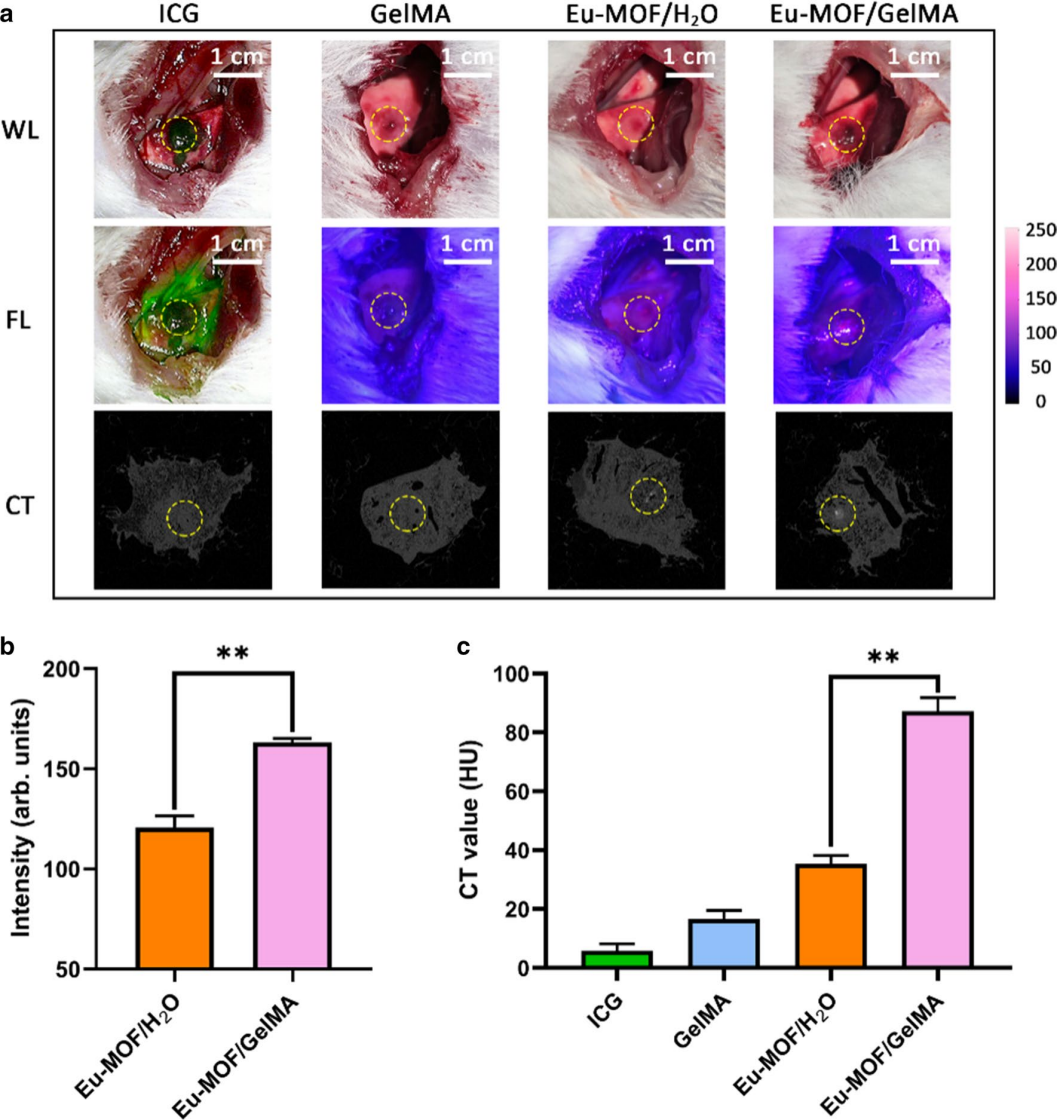
In vivo study of dual-modal imaging. **A** Fluorescence images and CT images of mouse lungs taken 2 h after the injection of an aqueous ICG solution, a GelMA solution, a Eu-MOF/H_2_O suspension, and a Eu-MOF/GelMA suspension. **B** Fluorescence intensities of the injection sites of the mouse lungs injected with Eu-MOF/H_2_O and Eu-MOF/GelMA suspensions. **C** CT values of the injection sites of the mouse lungs. **P < 0.01

As shown in the fluorescence imaging presented in Fig. 4A, the area of ICG diffusion was quite large across the mouse lung surface after 2 h, and even the parietal pleura was stained with green fluorescence. Likewise, the images recorded 2 h after injection of the Eu-MOF/H_2_O suspension show that the fluorescent area spread widely, and the fluorescence appeared to be too weak to be distinguished from the surrounding ordinary pulmonary parenchyma (Fig. 4A, B). However, the red fluorescence of Eu-MOF/GelMA was centralized at the injection point with a high fluorescence intensity. In addition, CT images of the Eu-MOF/GelMA samples showed an injection region with a prominently high density compared with those of the ICG, GelMA, and Eu-MOF/H_2_O systems (Fig. 4A). The CT values (HU) of the four groups were 5.789 ± 2.386, 16.662 ± 2.906, 35.357 ± 2.848, and 87.222 ± 4.636, for the ICG, GelMA, Eu-MOF/H_2_O, and Eu-MOF/GelMA systems, respectively (Fig. 4C).

## Discussion

In CT imaging, the normal pulmonary parenchyma has a relatively low density due to the large volume of gas contained in the lungs. Therefore, when early stage lung cancer occurs, the lesions are much easier to detect using CT imaging than other types of solid tumors [5]. As a result, the excision of pulmonary nodules has become one of the major operations carried out by thoracic surgeons worldwide [40, 41]. Since the lesions are quite small and sometimes even invisible and impalpable, the localization of pulmonary nodules is particularly challenging. As mentioned above, traditional dyes for their localization, such as ICG, exhibit a number of issues, such as rapid diffusion. The localization of deep-seated invisible and impalpable pulmonary nodules therefore remains a significant challenge when using ICG.

Thus, we attempted to address these issues by utilizing a MOF to enhance the available fluorescence. More specifically, using a Eu^3+^-based MOF and loading the luminescent material onto the hydrogel, Eu-UiO-67-bpy was successfully synthesized via a ligand-exchange reaction between Eu(TTA)_3_(BPY) and UiO-67-bpy. It should be noted that Eu^3+^ suffers from weak light absorption due to a forbidden 4f-4f transition, which renders the direct excitation of Eu^3+^ quite inefficient unless a high-power laser is employed. However, in the case of Eu-UiO-67-bpy, TTA and BPY were able to form stable structures surrounding Eu^3+^, in addition to playing important roles as organic ligands to sensitize the luminescence by overcoming the forbidden 4f-4f transitions. When under UV light radiation, the light is absorbed effectively by TTA and BPY, and the energy is transferred to Eu^3+^ from these ligands. Subsequently, luminescence is emitted by Eu^3+^; overall, this is known as the “antenna effect” [24, 42]. Since the peak emission was observed at 614 nm (i.e., in the visible spectrum), the luminescence could be seen even without the need for a specific optical device to detect fluorescence. Moreover, Eu^3+^ is known to efficiently absorb X-rays, thereby making it possible for Eu-MOF to be visible by CT imaging. With such an enhancement, surgeons can confirm the localization site immediately prior to the operation, as well as localizing deep-seated lesions using intraoperative CT systems.

For our hydrogel system, we selected GelMA as the liquid carrier, due to the fact that it is widely used in the biomedical field owing to its suitable biological properties and tunable physical characteristics [43]. A 90% substitution degree was selected for the amino group since this type of GelMA has a higher stiffness value compared with other GelMA species with lower substitution degrees. The concentration of GelMA was set at 18% to consider both the stiffness of the photocured solid hydrogel and the fluidity of the hydrogel solution. Thus, the hydrogel was expected to provide palpability for the injection site, thereby aiding the surgeons in detecting pulmonary nodules. Importantly, with this composition, the GelMA solution exhibited a suitable viscosity for injection when using fine needles. Indeed, the images of hydrogel blocks obtained under UV light excitation, in addition to the compressive modulus data, confirmed that the Eu-MOF/GelMA exhibited conspicuous red fluorescence, along with a substantially high stiffness. The biodistribution of high uptake by tumor compared to other important organs such as brain is regarded as an important property for nanoparticles when used for biomedical application [44, 45], as well as fast renal excretion of extra nanoparticles in the body [46, 47], but the cytotoxicity of nanoparticles is still concerned. Also, nanoscale materials can show some unexpected cytotoxic effects including ion release, even though the materials can be biocompatible in their bulk state [48, 49]. In our experiments, the biocompatibility of the Eu-MOF was confirmed using the CCK-8 assay. Besides, not only does the GelMA exhibit an excellent biocompatibility, but it also restricts the release of Eu-MOF into the surrounding tissue, and consequently reduces the overall toxicity of the Eu-MOF/GelMA complex [39]. And in clinical practice, the target lung segment with Eu-MOF/GelMA complex inside will be excised in a short time after the injection (usually less than 24 h). Therefore, both restricted content release and limited retention time minimize the biodistribution range in vivo and toxicity for other organs.

We then conducted in vitro and in vivo experiments on porcine lungs and mice, and it was found that the Eu-MOF/GelMA composite hydrogel exhibited a constant strong luminescence both in vitro and in vivo. In addition, it was found that the fluorescence diffusion area was restricted to the injection point compared with the cases of ICG and Eu-MOF/H_2_O due to the restriction provided by the crosslinked GelMA. Furthermore, the injection sites of the Eu-MOF/GelMA suspension could be clearly observed in the CT images, wherein relatively high CT values were obtained. It should also be noted here that the CT values of the various injectants were higher in the mouse lungs than in the porcine lungs. This may be explained by considering the dense interstitial space of mouse lungs, which limited diffusion of the solution or suspension. Moreover, deflation and removal of the lungs upon sacrifice of the mice likely compressed the liquid media. These results therefore demonstrate that our Eu-MOF/GelMA is suitable for using in the dual-modal imaging of pulmonary nodule localization.

However, we should point out some limitations to our research. Firstly, UV light has the ability to harm normal tissue when used for luminescence. Secondly, the recognition of the red fluorescence of Eu^3+^ could potentially be disturbed by the original red color of the surrounding tissue and blood. Thus, we wish to find an alternative material or method of modification to reduce any potential damage caused by radiation. Lastly, the mouse model employed for the in vivo experiment was not suitable for simulating the clinical operation, since the thoracic cavities were not sufficiently large for the endoscope to enter, and the mouse lungs were extremely small compared with those of humans, and so larger animal models should be investigated in the future.

## Conclusion

In summary, we synthesized a dual-modal contrast agent based on the Eu-UiO-67-bpy and proposed a strategy for the localization of small pulmonary nodules by the injection of GelMA loaded with the Eu-MOF. The fluorescent emission was constant and stable, and the diffusion of dyes was restricted to the injection point when using Eu-MOF/GelMA for localization. The complex was clearly visualized in the CT images, which enabled the dual-modal CT and fluorescence imaging of the injection sites. Moreover, the injection sites were highly tactile, thereby improving the accuracy of localization for surgeons during the operation. These results suggest that the use of this Eu-MOF/GelMA composite hydrogel may be a promising strategy for improving the accuracy of localizing and removing small pulmonary nodules.

## Methods

### Materials

Europium chloride hexahydrate, zirconyl chloride octahydrate, TTA, BPY, and 2,2′-bipyridine-5,5-dicarboxylic acid (BPY-DC) were purchased from J&K Scientific (Beijing, China). GelMA was purchased from Engineering For Life (Suzhou, Jiangsu, China), while ICG was purchased from the Dandong Yichuang Pharmaceutical Company (Dandong, Liaoning, China). HFL1 (Human fetal lung fibroblast 1) cells and HFL1 complete medium were kindly provided by the Stem Cell Bank of the Chinese Academy of Sciences (Shanghai, China). The trypsin–EDTA solution and phosphate-buffered saline (PBS) were purchased from Gibco (Grand Island, NY, USA).

### Synthesis of Eu(TTA)_3_(BPY)

Europium chloride hexahydrate (366 mg, 1 mmol), TTA (667 mg, 3 mmol), and BPY (156 mg, 1 mmol) were dissolved in a mixture of ethanol (5 mL) and water (1 mL). Subsequently, an aliquot (1 mL) of a 3 M NaOH solution was added to the above mixture to give a white precipitate. After continuous stirring at room temperature (25 °C) for 2 h, the reaction product was subjected to centrifugation, and the pellet was washed with a 1:1 mixed solvent of ethanol and water (10 mL), and dried prior to further use.

### Synthesis of UiO-67-bpy

BPY-DC (20 mg, 0.08 mmol) was added to DMF (1 mL) to give a white suspension. Zirconyl chloride octahydrate (9 mg, 0.028 mmol) was dissolved in DMF (3 mL) in a separate vial. Subsequently, these two solutions were mixed, and acetic acid (24 mL) was added. After brief sonication, the suspension was heated at 90 °C for 18 h to yield UiO-67-bpy.

### Synthesis of Eu-UiO-67-bpy (Eu-MOF)

UiO-67-bpy (50 mg) and Eu(TTA)_3_(BPY) (30 mg) were added to DMF (5 mL), and after ultrasonic dispersion for 5 min, the mixture was sealed and heated in an oven at 120 °C for 3 h, and then cooled to room temperature. Finally, the product was subjected to centrifugation, washed with a 1:1 mixed solvent of DMF and ethanol (20 mL), and dried prior to further use.

### Physical characterization of Eu-MOF

TEM images and the corresponding mapping images were obtained using a JEM-2100 field emission transmission electron microscope (JEOL, Tokyo, Japan). The XRD patterns were obtained using a D8 Advance instrument (Bruker, Karlsruhe, Germany). The fluorescence spectra were obtained using an FS5 Spectrofluorometer (Edinburgh Instruments, Livingston, UK).

### Biocompatibility of Eu-MOF

HFL1 cell suspensions were seeded into 96 well plates at a density of 5 × 10^3^ cells per well and incubated overnight under standard conditions (37 °C, 5% CO_2_). Subsequently, the cells were incubated with different concentrations of Eu-MOF (0, 0.05, 0.1, 0.25, 0.5, and 1 mg/mL) for 24 h. After this time, each well was washed three times with PBS to remove any residual Eu-MOF, and then an aliquot (100 μL) of the HFL1 medium and the CCK-8 solution (10 μL) were added. After incubation for 2 h, the absorbance at 450 nm was measured, and the relative cell viability was calculated.

### Preparation of the Eu-MOF/GelMA composite hydrogel

GelMA (1 g) was added to the photoinitiator solution (5 mL) and heated at 65 °C in a water bath protected from light to obtain a 20% (w/v) GelMA solution. The GelMA solution was filtered using a syringe filter with a 0.22 µm pore size prior to further use. The aqueous Eu-MOF suspensions (0.1 mL) with concentrations of 0, 12.5, 25, 50, 100, and 200 mg/mL were mixed with the GelMA solution (0.9 mL), and the Eu-MOF/GelMA mixtures of different concentrations were poured into molds with an internal diameter of 5 mm and a height of 2 mm. After irradiation with 405 nm light for 30 s, the photocured solid hydrogels were separated from the molds.

### Physical characterization of the composite hydrogel

The fluorescence images of the Eu-MOF/GelMA composite hydrogel were recorded under irradiation with 365 nm UV light, and the fluorescence intensity of each hydrogel block was quantified using the ImageJ software. CT scans and 3D reconstructions were performed using a μCT 80 Micro-CT System (SCANCO Medical, Bassersdorf, Switzerland). The mechanical characteristics were tested using an Instron 5542 dynamic mechanical analysis instrument (Canton, MA, USA). The compressive modulus was calculated using the slope of the linear region in the 0–10% (0–0.2 mm) strain range of the stress–strain curves.

### In vitro study

The aqueous ICG solution (2.5 mg/mL), the GelMA solution (18%), the Eu-MOF/H_2_O suspension (Eu-MOF aqueous suspension, 5 mg/mL), and the Eu-MOF/GelMA suspension (mixture of 0.1 mL of the 50 mg/mL Eu-MOF suspension and 0.9 mL of the 20% GelMA solution) were prepared previously. Lung segments of similar sizes were separated from porcine lungs. The lung segments were then injected with one of the four solutions (0.1 mL), and were then irradiated with 405 nm light for 30 s. White light images and fluorescence images of the lung segments were taken before injection, and then 5 min and 2 h after injection. Fluorescence images of segments with ICG injection were captured using an Optomedic 2100 Series HD Fluorescence Endoscopic System (Foshan, Guangzhou, China). The micro-CT images and CT values were obtained 2 h after injection. Additionally, a further two groups of lung segments were injected with Eu-MOF/H_2_O and Eu-MOF/GelMA for 24-h observation of their fluorescence.

### In vivo study

Six-week-old BALB/c mice were anesthetized via the intraperitoneal injection of a chloral hydrate solution (10 wt%; 5 mL/kg) and placed in the right lateral decubitus position. The lower edge of the rib cage was identified, the fur in the area was shaved, and the incision site was sterilized with 75% ethanol. A 1 cm incision was made through the skin subcutaneous tissue to reveal the rib cage along the line 1 cm superior to the lower edge of the rib cage. Subsequently, incisions of 0.5 cm were made of the intercostal muscle. The left lungs were injected with an aliquot (0.05 mL) of the aqueous ICG solution, the GelMA solution, the Eu-MOF/H_2_O suspension, or the Eu-MOF/GelMA suspension, and were then irradiated with 405 nm light for 30 s. Following irradiation, the incisions were sewn up immediately, and the redundant gas in the thoracic cavity was drained using a syringe to maintain a low pressure inside the cavity. The mice were sacrificed 2 h after the injection. White light images and fluorescence images were obtained after the removal of some of the ribs. Finally, the lungs were separated and sent for micro-CT scans.

### Statistical analysis

All quantitative data are presented as the mean ± standard deviation. Data were analyzed using GraphPad Prism 8.0, and the statistical significance was set at P < 0.05.

## Supplementary Information

**Additional file 1: Fig. S1**. Test of the Tyndall effect for the GelMA, Eu-MOF/H_2_O, and Eu-MOF/GelMA suspensions.

**Additional file 2: Fig. S2**. Fluorescence images of a 72-h observation of porcine lung segments injected with Eu-MOF/H_2_O and Eu-MOF/GelMA.

**Additional file 3: Video S1**. Palpability test of the Eu-MOF/GelMA suspension injection site of porcine lung.

## Data Availability

The data described in the manuscript are available from the corresponding author on reasonable request.

## Abbreviations

ICG: Indocyanine green
MOFs: Metal–organic frameworks
UiO: University of Oslo
bpy: 2,2'-Bipyridyl
BPY: 2,2'-Bipyridine
GelMA: Gelatin-methacryloyl
CT: Computed tomography
NIR: Near-infrared
UV: Ultraviolet
TTA: 2-Thenoyltrifluoroacetone
TEM: Transmission electron microscopy
XRD: X-ray diffraction
CCK-8: Cell counting kit-8
BPY-DC: 2,2′-Bipyridine-5,5-dicarboxylic acid
