# Supplementary material for: Lanthanide-based metal–organic frameworks solidified by gelatin-methacryloyl hydrogels for improving the accuracy of localization and excision of small pulmonary nodules
Source: J Nanobiotechnology. 2022 Feb 2;20:60. doi: 10.1186/s12951-022-01263-6 (PMC8808773; doi:10.1186/s12951-022-01263-6)
Supplement: Supplementary file 1 — Additional file 1: Fig. S1. Test of the Tyndall effect for the GelMA, Eu-MOF/H2O, and Eu-MOF/GelMA suspensions. [file 12951_2022_1263_MOESM1_ESM.docx]

Supporting Information

**Lanthanide-Based Metal–Organic Frameworks Solidified by Gelatin-Methacryloyl Hydrogels for Improving the Accuracy of Localization and Excision of Small Pulmonary Nodules**

*Haoran Ji^1#^, Xiaofeng Wang^2#^, Pei Wang^1^, Yan Gong^1^, Yun Wang^1^, Guangyu Ji^1*^, Chang Liu^1^, Xiansong Wang^1*^, and Mingsong Wang^1*^*


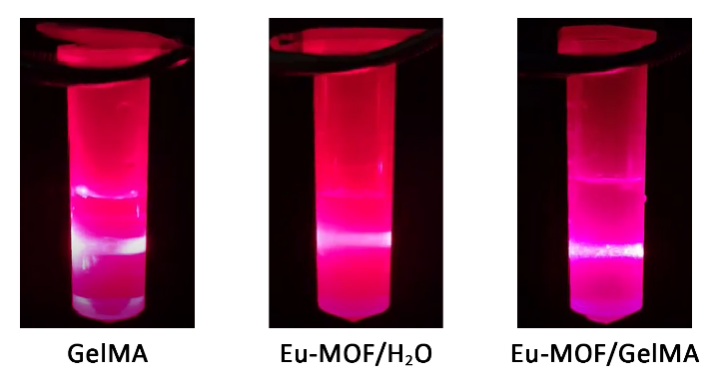


**Fig. S1** Test of the Tyndall effect for the GelMA, Eu-MOF/H_2_O, and Eu-MOF/GelMA suspensions.
