# Supplementary material for: Lanthanide-based metal–organic frameworks solidified by gelatin-methacryloyl hydrogels for improving the accuracy of localization and excision of small pulmonary nodules
Source: J Nanobiotechnology. 2022 Feb 2;20:60. doi: 10.1186/s12951-022-01263-6 (PMC8808773; doi:10.1186/s12951-022-01263-6)
Supplement: Supplementary file 2 — Additional file 2: Fig. S2. Fluorescence images of a 72-h observation of porcine lung segments injected with Eu-MOF/H2O and Eu-MOF/GelMA. [file 12951_2022_1263_MOESM2_ESM.docx]

**
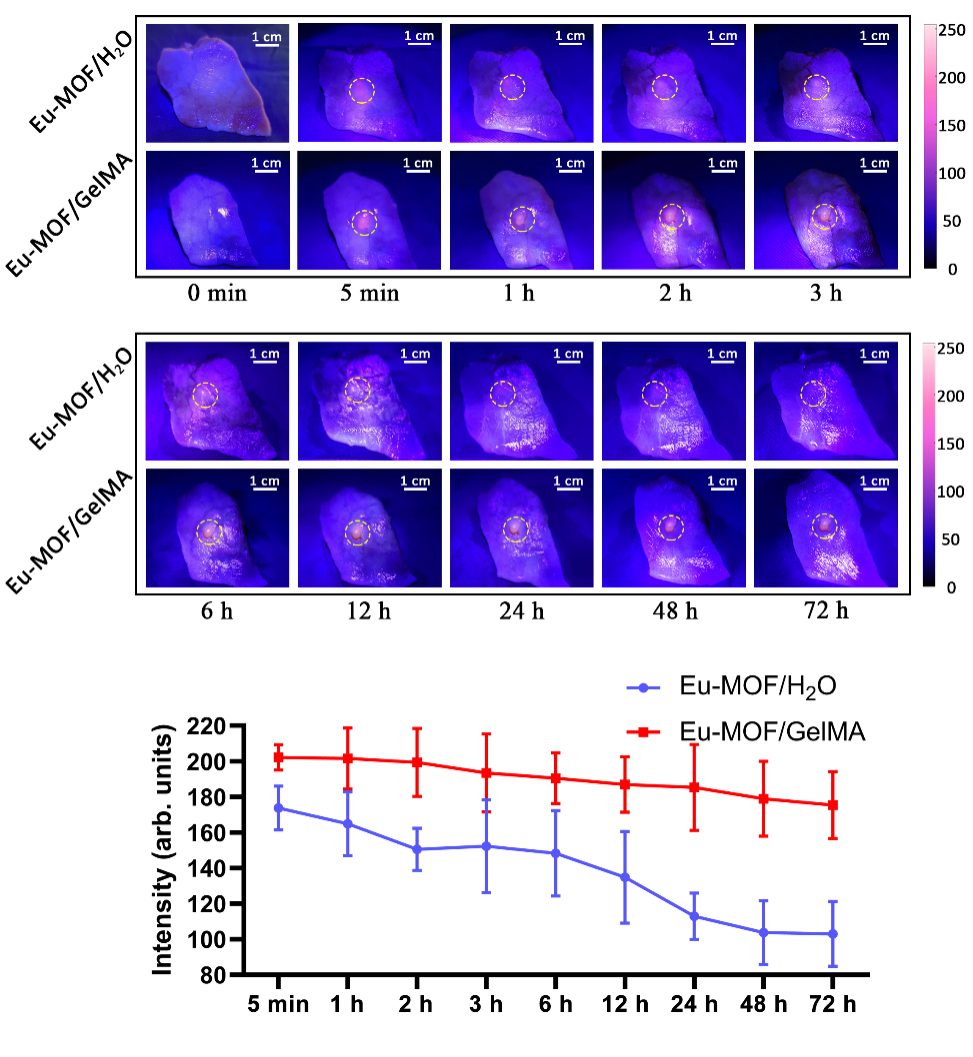
**

**Fig. S2** Fluorescence images and fluorescence intensity variations of a 72-h observation of porcine lung segments injected with Eu-MOF/H_2_O and Eu-MOF/GelMA suspensions.
